# Integrating Preclinical and Clinical Models of Negative Urgency

**DOI:** 10.3389/fpsyt.2019.00324

**Published:** 2019-05-23

**Authors:** Meredith Halcomb, Evangelia Argyriou, Melissa A. Cyders

**Affiliations:** ^1^Department of Radiology and Imaging Sciences, Indiana University School of Medicine Indianapolis, Indianapolis, IN, United States; ^2^Department of Psychology, Indiana University–Purdue University, Indianapolis, IN, United States

**Keywords:** negative urgency, animal model, delay discounting, Impulsive behavior, internalizing disorder, transitional, UPPS Impulsive Behavior Scale

## Abstract

Overwhelming evidence suggests that negative urgency is robustly associated with rash, ill-advised behavior, and this trait may hamper attempts to treat patients with substance use disorder. Research applying negative urgency to clinical treatment settings has been limited, in part, due to the absence of an objective, behavioral, and translational model of negative urgency. We suggest that development of such a model will allow for determination of prime neurological and physiological treatment targets, the testing of treatment effectiveness in the preclinical and the clinical laboratory, and, ultimately, improvement in negative-urgency-related treatment response and effectiveness. In the current paper, we review the literature on measurement of negative urgency and discuss limitations of current attempts to assess this trait in human models. Then, we review the limited research on animal models of negative urgency and make suggestions for some promising models that could lead to a translational measurement model. Finally, we discuss the importance of applying objective, behavioral, and translational models of negative urgency, especially those that are easily administered in both animals and humans, to treatment development and testing and make suggestions on necessary future work in this field. Given that negative urgency is a transdiagnostic risk factor that impedes treatment success, the impact of this work could be large in reducing client suffering and societal costs.

## Introduction

Negative urgency is an impulsive personality trait reflecting the tendency to act rashly when experiencing extreme negative emotional states, included in the UPPS-P Model of Impulsive Behavior (1, 2). Overwhelming evidence suggests that negative urgency is robustly associated with rash, ill-advised behavior, and this trait may hamper attempts to treat patients with substance use disorder [e.g., Refs. (3, 4)]. However, a systematic investigation of negative urgency in the context of treatment has been limited, in part, due to the lack of a valid objective, behavioral, and translational model of negative urgency. The goal of the current paper is to review the current human and animal approaches to the measurement of negative urgency and to make suggestions on how an objective translational model could be developed. We review the existing literature and make suggestions for prime models that can be explored as translational approaches in negative urgency. We also review the neural and psychopharmacological correlates of negative urgency, suggesting potential novel targets of intervention within a translational model. We suggest that the development of a translational model easily administered in animals and human would allow for better characterization of the neuroscientific correlates of negative urgency, determination of prime neurological and physiological treatment targets, and the validation of an objective measure of treatment effectiveness in the preclinical and clinical laboratory.

## Negative Urgency in The Broader Construct of Impulsivity and Personality

Impulsivity is broadly defined as traits and behaviors that predispose individuals to rash action (or ill-advised inaction) (5–7). The UPPS-P model integrated existing personality-based measures of impulsivity into five traits, using the Five-Factor Model as a theoretical framework. The five traits include negative urgency, positive urgency (i.e., a tendency to act rashly in response to extreme positive emotional states), lack of premeditation (i.e., a tendency to act without thinking), lack of perseverance (i.e., an inability to stay focused on a task), and sensation seeking (i.e., tendency to seek novel and exciting experiences). These traits are best described as separate, though related, tendencies toward rash action (8). Research supports a multidimensional nature of impulsivity, and extensions of the UPPS-P model have been suggested (9). There is increasing consensus that impulsive personality consists of traits that are affect-free and traits that have a strong affective component (9, 10). The distinction between affect-based and affect-free impulsigenic traits is further supported by the fact that they share little common variance (0–13%) (10).

Negative urgency is well placed in the personality literature. It shares conceptual overlap with the Impulsiveness facet of the NEO-PI-R (11); however, a factor analysis by Peterson and Smith (2008) found that negative urgency loaded onto the Neuroticism, Conscientiousness, and Agreeableness factors, suggesting that negative urgency is not represented by one domain or facet of the NEO-PI-R, but rather assesses a trait characterized by high distress, low conscientiousness, and low agreeableness (2). Some have suggested that negative urgency (along with the positive mood variant of positive urgency) is quite similar to one of the two higher-order dimensions of the Five-Factor Model (alpha, representing high levels of emotional instability, disagreeableness, and disinhibition) (12, 13).

What differentiates negative urgency from other constructs pertaining to responses to emotions, such as emotion regulation and emotional lability, is that it reflects a disposition to reflexive reactions in response to intense negative emotion. Emotion regulation involves efforts, either reflexive or effortful, to modify the intensity of the experienced emotion that varies across situations and across time, and emotion dysregulation can occur in the absence of intense emotion (14, 15). Negative urgency captures the between-person variability in the capacity to control intense emotion-driven urges (10). Effects of negative urgency are not explained by additive or interactive combinations of negative affective traits (e.g., neuroticism, emotional lability) combined with general disinhibition (2, 8). Similarly, negative urgency is only moderately correlated with measures of emotion regulation, which signifies that these are related, but separate, constructs with distinct contributions to psychopathology (16). In fact, the majority of the reliable variance in negative urgency is not explained by other related traits (2).

## Negative Urgency as a Transdiagnostic Risk and Treatment Factor

Accumulating evidence suggests that negative urgency is one of the most robust predictors of a wide range of maladaptive behaviors and psychopathology, including alcohol use and dependence (3, 5, 17, 18), tobacco use and dependence (19–21), and problematic cannabis use (22–24). The fact that negative urgency developmentally precedes substance use and addictive disorders (25, 26) indicates that negative urgency is likely a contributor to the development and maintenance of addictive disorders. This is further bolstered by empirical evidence showing that decreases in impulsivity are associated with decreases in substance use across the lifespan (27). This accumulating evidence supports the notion that negative urgency is a transdiagnostic endophenotype for problematic levels of behaviors associated with risk (28). This includes not only addictive behaviors, but also disorders highly comorbid with these conditions, such as depression, anxiety, and bipolar disorders (5, 28, 29). Negative urgency is represented in broad diagnostic criteria of the *Diagnostic and Statistical Manual of Mental Disorders, Fifth Edition* (*DSM-5*) (29).

Despite the substantial amount of research implicating negative urgency in the development and maintenance of addictive behaviors, only a small body of empirical work has systematically studied its application to treatment (29, 30). There are no specific behavioral or pharmacological treatments for negative urgency, although some have been suggested (31). A meta-analysis conducted by Hershberger et al. (30) examined the effect of negative urgency on substance use disorder psychotherapy outcomes and how this trait changes during treatment. The findings showed that increased levels of negative urgency at baseline are related with poorer treatment outcomes, suggesting that this trait potentially inhibits substance use symptom improvement (30). Additionally, the authors identified only small decreases in negative urgency (*g* = −0.25) from the beginning to the end of treatment. This suggests that current substance use treatments are not changing negative urgency notably, which increases the risk for subsequent substance use re-initiation or relapse (30). They explain one way in which negative urgency lowers treatment efficacy: Most existing therapies for addictive disorders are focused on the modification of proximal factors related to addiction, such as substance use motives or environments that facilitate use, rather than the distal factors, such as negative urgency, that underlie them (32, 33). For example, negative urgency is a predictor of the development of substance use motives (34) and likely contributes to individuals seeking out and selecting environments that facilitate use, consistent with personality–environment transaction theories (35). Although addressing proximal risk factors of addiction might improve current symptoms, if distal risk factors remain unchanged, relapse or treatment nonresponse becomes more likely (29), as the distal factors can impart risk independent of the modified proximal factor. The authors suggest that the integration of negative urgency in case conceptualization, treatment planning, and goal setting would significantly improve substance use treatment outcomes (30).

Although negative urgency-targeted interventions have not been systematically developed or investigated, there is promising evidence for their potential success. Zapolski et al. (31) provided recommendations for strategies to target negative urgency in treatment. Their recommended strategies include training in emotion regulation, distress tolerance, interpersonal effectiveness, training in modifying emotional reactions based on the context, relaxation techniques, identification of precipitating events and triggers to emotional reactivity and use of adaptive alternatives, and the use of medications, such as selective serotonin reuptake inhibitors (31, 36). Many of these strategies have been successfully incorporated in several clinical interventions in different contexts, including substance use, and their effectiveness has been tested and supported (5, 26, 36–40) with some exceptions (41, 42). Because negative urgency increases the risk of a wide range of addictive behaviors and other clinical disorders, negative-urgency-targeted interventions could have wide and broad benefit. Additionally, such interventions are easily adopted by addiction medicine practitioners and would improve their daily practice in prevention, treatment, and rehabilitation of addictive disorders and accompanying conditions.

We propose that an important and viable long-term goal is to design and test pharmacological, psychological, behavioral, and physiological treatments that specifically aim to reduce negative urgency. This would allow the application of these treatment strategies transdiagnostically, which would be fruitful to reduce not only the target disorder (e.g., alcohol use disorder), but also maladaptive coping related to comorbid disorders (e.g., depression). Thus, one intervention could be effective for treatment of multiple disorders or behaviors. In the current paper, we focus specifically on the role of negative urgency in addictive disorders, although the implications would likely apply to any disorder in which negative urgency is implicated (29).

## Current Measurement of Negative Urgency in Humans

Negative urgency is most commonly measured using the UPPS-P Impulsive Behavior Scale. The UPPS-P is a 59-item self-report questionnaire originally created by Whiteside and Lynam (1) with four subscales (negative urgency, lack of premeditation, lack of perseverance, and sensation seeking). The positive urgency subscale was added later (43, 44). Individuals rate their general tendencies on a four-point scale from *Agree Strongly* to *Disagree Strongly*. Individual item scores are reverse coded (as needed) and averaged together to approximate a mean level of each trait, with higher scores indicating higher levels of rash action. The negative urgency subscale includes items assessing the disposition of respondents to act without careful consideration of the consequences when faced with negative affect. Example items for the scale include “When I feel bad, I will often do things I later regret in order to make myself feel better now” and “When I am upset, I often act without thinking.” The UPPS-P has been shown to produce valid and reliable data across men and women, different age groups, and clinical and community samples (43, 45–47).

Although there are no behavioral tasks developed specifically to assess negative urgency, there are many behavioral tasks measuring state-like rash action in general (48, 49). Both self-report and behavioral task measures of rash action are strongly correlated with risky behaviors, but research has shown little overlap *between* these two types of measures. A meta-analysis conducted by Cyders and Coskunpinar (48) found that effect sizes for the relationship between self-report and behavioral task measures of rash action are small, ranging from *r* = 0.097 to 0.134, suggesting that at least 99% of the variance between these types of measures is unshared. This indicates that self-report and behavioral measures of rash action assess complementary, but separate, constructs. In some ways, this lack of overlap is not surprising, as these different task domains assess separate aspects of rash action. Self-report measures likely represent stable tendencies toward general behaviors (trait-like impulsivity) reflecting predominately emotional/motivational mechanisms of rash action, whereas behavioral tasks are more likely snapshots of behavior (state-like impulsivity) in response to stimuli, reflecting predominately cognitive mechanisms of rash action (9).

Self-report measurement of negative urgency has important strengths in the context of psychopathology. First, it has content and ecological validity as it reflects the individuals’ subjective experience of patterns of thoughts, emotions, and behaviors in daily life, which can easily be generalized to the real world (50). Second, it has strong face validity, in that questions and results can be easily interpreted without making assumptions since they are based on direct and clear questions to the respondents (29, 48). Finally, it is inexpensive and easy to administer to a large group of people (48). However, this type of assessment has some limitations that make it less ideal in designing or testing the efficacy of treatments. Self-report measurement is limited by self-awareness, openness, and social desirability (48, 51), making the assessment only as good as what a person knows and is willing to report about the self. For example, in a clinical trial, individuals might not report on less socially desirable aspects of the self, and thus baseline levels might be underestimates of the true level of negative urgency, making measuring change over the trial more difficult. Relatedly, measuring negative urgency repeatedly in short succession might lead to participant fatigue or might influence an individual’s responses in undue ways, further contributing to error in the measurement. For example, in a clinical trial, individuals might report a reduction in negative urgency after treatment, because they assume such a reduction is expected and not due to any true changes in the trait in response to treatment. Additionally, because the UPPS-P evaluates general tendencies, changes that do occur in shorter time frames might not be accurately assessed *via* this measure (i.e., it is not designed to assess shorter fluctuations in behavior). Finally, self-report measures are difficult to translate into animal models. Behavioral tasks designed to measure the behavioral expression of negative urgency in lab settings would be an excellent complementary approach to address these limitations and to better design and assess treatment effectiveness.

## Animal Models of Negative Urgency

The use of animal models would greatly enhance the capability to deconstruct possible underlying neural mechanisms of negative urgency and allow for greater manipulation of testing variables to determine new therapeutic targets. There are numerous papers describing external validity, including the specific criteria animal models must meet and how different facets of external validity simulate conditions, neurobiology, and behavior seen in clinical populations (e.g., 52–55). The earliest characterization of these criteria included the requirement for animal models to demonstrate the same etiology, symptoms, response to treatment, and biochemistry seen in human populations (54). These criteria were the foundation for decades of work establishing a well-defined framework for animal models. The traditional framework, proposed by Willner (55), included three types of validity: *predictive validity* (i.e., the model predicts some criterion of interest), *face validity* (i.e., the model looks similar to the human condition), and *construct validity* (i.e., the animal model measures what is intended to measure) (55). Expansions and modifications of these criteria provide a more granular method for establishing reliable, translatable animal models, taking into account several factors that represent critical points for similarity. Geyer and Markou (53) emphasized the additional importance of *etiological validity* (i.e., the model has the same etiology as human condition) and *convergent/discriminant validity* (i.e., the model is associated with other related models but unrelated to models that are disparate with the underlying condition). Belzung and Lemoine (52) further emphasized *induction validity* (i.e., etiological effects on observable behaviors in animals have similar effects in humans) and *remission validity* (i.e., similarity in response to treatment across animal and human conditions).

Any animal model of negative urgency must fulfill these validity requirements; we evaluate existing and potential models in terms of these criteria (**Table 1**). For the sake of simplicity, we have not included every measure, but rather those we believe most strictly comply to a model of negative urgency. We also highlight the types of validity each model satisfies, where applicable, which aids in determining translatability.

**Table 1:** Methods for induction of negative affect and measurement of impulsive responding. This table lists several possible suggestions for induction of negative affect in animals and humans and impulsivity assessment. It also outlines which types of validity (which describes translatability) are fulfilled with each task. NEO-PI-R: NEO Personality Inventory, revised; IAPS: International Affective Picture System; na: no available data.

TASK	APPLICABLE IN HUMANS	APPLICABLE IN ANIMALS	TYPES OF VALIDITY					
			FACE	CONSTRUCT	ETIOLOGICAL	CONVERGENT	INDUCTION	REMISSION
INDUCTION OF NEGATIVE AFFFECT								
REWARD OMMISSION TASK	+	+	+	+	+	+	+	na
IAPS	+							
Paced Auditory Serial Addition Test	+							
IMAGINATION Mood Induction Procedure	+							
VELTEN Mood Induction Procedure	+							
SOCIAL REJECTION (humans)	+							
SOCIAL ISOLATION (animals)		+		+			+	na
FOOT SHOCK		+		+				na
FOOD DEPRIVATION		+		+			+	na
ACUTE RESTRAINT STRESS		+		+			+	na
MEASURES OF IMPULSIVITY								
DELAY DISCOUNTING	+	+	+	+	+	+	+	+
GO/NO-GO	+	+	+	+	+	+	+	+
STOP SIGNAL	+	+	+	+	+	+	+	na
CONTINUOUS PERFORMANCE TASK	+	+	+	+	+	+	+	na
BALLOON ANALOGUE RISK TASK	+			+				na
ERIKSEN FLANKER TASK	+							
SELF-REPORT MEASURES								
UPPS-P	+					+		
NEO-PI-R (Impulsiveness facet)	+					+		

At present, studies investigating negative urgency in animals are sparse. One proposed method for generating negative urgency (utilized in both animal and human models) involves unexpected reward omission (56–58). In one study, humans or rats were trained to perform an operant task (button pressing or lever pressing, respectively) and were rewarded with either money or a food pellet, respectively (56). Increases in response rates and decreases in response latencies were dependent variables constituting measures of negative urgency. This task has many strengths, including that it can and has been applied across human and animal models and that it appears to have adequate predictive and face validity for the emotional change. The task is especially analogous in clinical and preclinical administration (56); however, this task lacks the “rash action” component necessary to accurately assess negative urgency. For the model to have good external validity, there must be some procedure in place to assess the effect of this induced negative urgency on impulsive behavior. In short, this model does not provide any negative consequences of impulsive behavior generated through negative affect. Therefore, although this task shows some promise, further research is required for better, more representative models.

## Suggestions for Objective Translational Models of Negative Urgency

### Affective State

Clinical designs for incitement of negative affect in humans include the International Affective Picture System (IAPS), the Paced Auditory Serial Addition Test (PASAT), and social rejection, although there are no analogous methods in animal models (59, 60). The IAPS involves presentation of positive, neutral, or negative images (61). Numerous studies have shown that the administration of the IAPS is effective in producing transient negative emotion with resultant changes in brain activity or behavior (62–66). However, none of these studies has demonstrated increased impulsive, rash behavior associated with that negative affect. The PASAT is a procedure in which subjects must serially add a quickly vocalized list of numbers and is a demonstrated lab-induced stressor (67). Implementation of a social rejection protocol has generated changes in response inhibition during a Go/No-Go task (64, 68), thus linking negative affect with rash action. A meta-analysis by Westerman (69) outlines numerous other mood induction paradigms (including video clips, writing, etc). and describes the strengths and drawbacks of each. For example, the Imagination mood induction procedure requires the subject to imagine an emotion-laden experience from their past, and the Velten mood induction procedure requires the participants to make negative self-references (69). Unfortunately, these methods are not conducive to reproduction with animal models.

Although these findings suggest usefulness of these methods in humans, translatability to animals is questionable at best. There is no comparable method to many of these procedures (IAPS, PASAT, Imagination, Velten) in preclinical studies. While social isolation models in rodents can elicit behavioral modifications, these are typically utilized to produce depressive states and may instigate neurobiological alterations that have little connection to impulsivity and may hinder the ability to interpret results (54, 70, 71). For greatest translatability, the methods by which negative urgency are elicited should be as similar as possible in human and animal subjects.

Currently, there are several animal models capable of inducing stress, anxiety, and depression, such as restraint stress, foot shock, and the forced swim test (72–75). Many of these suffer from limited translatability to clinical models, due to ethical or feasibility restraints. Additionally, these models typically result in long-term effects (including downstream effects on neurotransmitter systems), in addition to immediate, negative states. For an animal model to have good face and etiological validity, there must be some instigating event that engenders a transitory negative state without conferring semi-permanent or permanent change. Given the effects of corticosterone on numerous brain regions, including the hippocampus (76, 77), the task should avoid chronic stressors and focus on events that primarily lead to depressive-like states. Additionally, it is important to limit exposure to the negative stimulus to avoid creation of a disposition to depression often seen after repeated administration (78). This distinction permits researchers to narrow investigations to discrete elicited state-like behavior rather than long term, trait-like behavior.

### Impulsive Choice

Delay discounting is based on the premise that reinforcer influence on behavior decreases as a function of the delay to its delivery (79). In one version of this task, the adjusting amounts version, subjects complete several trials in which they must choose between a small, immediate reward and a larger, delayed reward and every choice of the immediate reinforcer decreases the amount of reinforcer available upon choice of the immediate reinforcer on the next trial (80, 81). In this manner, repeated choice of the immediate reinforcer results in overall suboptimal levels of reinforcer across the session. Although delay discounting is typically thought to assess levels of cognitive impulsivity (82), the design of the task is such that impulsive-like responding is rewarded immediately (immediate reinforcer) and is then consequently paired with decrease in immediate reinforcer volume. Through manipulation of the length of the delay to the larger reward and the use of the hyperbolic discounting equation, we are able to generate a “discounting curve” that describes the steepness or “impulsivity” of each individual. This curve can be thought of as a measure of how long the subject is willing to wait for a specific reward, a measure of “cognitive impulsivity.”

Previous work has demonstrated the translatability of delay discounting. The delay discounting model has considerable face validity (52). The basic premise is identical in both human and animal models, particularly in the Experiential Discounting Task for humans, which requires the participant to experience the delay during the task, rather than afterwards, eliminating the need for the subject to “imagine” the delay while continuing to respond (83). In the animal version, subjects are also required to experience the delay during the session. Furthermore, one of the proposed mechanisms of external validity is the requirement that the task must measure the same changes in behavior upon treatment (remission validity) (52). Although this version of delay discounting most closely resembles the version administered in animals, there are possible limitations in test–retest reliability and conflating the delay and probability of receiving reward (84). A more common administration (the adjusting amounts version) involves presenting a delayed choice that will be accessible at some point in the future rather than implementing the delay during the task itself. The adjusting amounts version is also efficient at generating discounting curves and there is no demonstrable difference in effect between delayed rewards during the task and those imagined in the future (85). The animal version of delay discounting described here is accurate enough to detect the same decreases in impulsivity after stimulant (methamphetamine/amphetamine) administration observed in clinical applications (86–91). Given the demonstrated ability of the delay discounting task to evaluate changes in impulsive, rash behavior, and the translatability of those results, it may provide a valid mechanism to analyze behavior motivated by a negative emotional state.

### Impulsive Action

In the Go/No-Go task, the subject is required to respond on a specified manipulandum upon presentation of some stimulus during “Go” trials and must inhibit that response upon presentation of some stimulus during “No-Go” trials (92, 93). This method is used to measure action restraint in a number of animal models of disease, including alcohol use disorder (94). A meta-analysis found a significant correlation between results on clinical applications of the task with self-report measures of negative urgency (48). A recent study examining the association between induced negative urgency and performance on the Go/No-Go task revealed that greater activation in brain regions involved in inhibitory processes was correlated with higher levels of urgency (64–66, 68). An investigation into the effects of social rejection on impulsivity found that subjects reporting higher levels of negative affect completed significantly fewer successful No-Go trials (95). There is also evidence that responding in the Go/No-Go task predicts relapse rates in abstinent alcoholics (96). Administration of this task in an animal model of negative urgency could pave the way toward understanding what neural correlates underlie this association.

### Conclusions on Translational Tasks

In conclusion, although the literature on animal models of negative urgency is sparse, there are some interesting and promising attempts to model negative urgency preclinically. The very nature of negative urgency centers upon behavioral reactions to emotional states, suggesting an internalizing primary aspect of the trait that is integrated with an externalizing behavioral outcome. Therefore, any reliable, translatable model of negative urgency must include a method for inducing negative affect in addition to the demonstration of an externalizing behavior of some interest. Unfortunately, the design of preclinical models of internalizing affective disorders is inherently problematic. Emotional states, such as depression, are not easily represented in non-human subjects, which limits the ability to devise translational, behavioral measures (52, 78, 97–99). Any animal model seeking to evaluate affectivity must demonstrate the capacity not only to induce a specific emotional state, but also to effectively identify alterations in that state under manipulation. Since evaluating the subjective experiences of animals is difficult, this is a challenging prospect; however, the translatability of the model is necessary for the generation of meaningful results (52).

Ideally, the behavior evoked by the induction of negative affect should be immediately reinforcing, yet ultimately yield suboptimal results. Cyders and Smith (100) proposed that rash or ill-advised actions during times of negative arousal, such as consuming alcohol upon receipt of bad news, provide immediate relief, reinforcing the behavior (100). Alternatively, more adaptive coping mechanisms are not implemented, limiting the reinforcement of these responses. For example, then, engagement in repeated alcohol intake to alleviate negative affectivity is reinforced and the behavior becomes more frequent, despite being detrimental in the long term. The key in an animal model is to devise a task that provides an opportunity to access a preferred reward (food, sucrose, mating), paired with loss of a highly valued resource or punishment, such as excessive lever pressing, resulting in smaller amounts of reward over time.

## Neural and Genetic Correlates of Negative Urgency and Potential Treatment Targets

There are numerous excellent reviews outlining human brain structures and systems believed to contribute to the experience of negative urgency (2, 101) and other emotion-related constructs (9, 102–104). Given the implicit obstacles associated with neuroimaging in rodents and the lack of definitive homologous prefrontal regions in rodent brains, there has been limited information gleaned from animal neuroimaging studies.

### Human Neuroimaging

Research concerning the neurobiology underlying negative urgency follows two primary tracts: structures and systems that represent bottom-up processing and structures and systems that are associated with top-down regulatory control. Regions representing bottom-up processing, such as the amygdala, have connections to regions regulating top-down control, including the orbitofrontal cortex (OFC), the ventral medial prefrontal cortex (vmPFC), and the dorsolateral PFC (dlPFC). These connections constitute a reciprocal system by which both systems integrate to detect negative affect, determine the salience of that affect, and initiate or inhibit behavior in reaction (2). For instance, the amygdala is greatly involved with the experience of negative emotions (105) and projects to, and receives back projections from, the OFC (106). The OFC and its medial sector, the vmPFC, play a role in modulating emotion-based behavior and reactivity (105) and can inhibit behavior that is emotion-based (107). The ability of top-down regulatory systems to control emotion-based behavior is essential for preservation of long-term goals. There is emerging evidence that negative urgency relates to variations in neural activity in these and other subcortical [ventral striatum (VST) and caudate nucleus (CAU)] and frontal (dlPFC) brain regions (57, 64, 68, 108, 109).

There is a wealth of information concerning the function of these brain regions from neuroimaging studies, implicating the association of various structures in negative-urgency-mediated ill-advised behaviors, particularly in alcohol use disorder populations and social drinkers. The insula (INS), which plays a role in emotional processing, shows greater activation during negative urgency in adolescent binge drinkers (110). The caudate (CAU) demonstrates greater activation in response to alcohol cues in social drinkers (64, 68). The mOFC/vmOFC, primarily engaged in evaluation of rewarding stimuli to determine action, is activated more strongly to alcohol odor cues, while activation of the lOFC, which evaluates punishing stimuli, was related to negative urgency in social drinkers (63). The PFC, particularly the dlPFC, is heavily recruited during cognitive tasks in subjects high in negative urgency (111, 112). Finally, the amygdala, which is specifically involved in processing negative emotions (113) and is an important hub in negative urgency (101), shows greater activation in response to negative mood images and during negative mood evaluation in cocaine users with personality disorders (114, 115). Taken together, these findings imply a dysregulation in this interconnected system of regions associated with emotional processing and emotion-based behavioral control, which is likely contributing to maladaptive actions in pathological populations.

Emerging evidence from functional connectivity studies indicates that these structures interact to direct emotional lability and drive behavior. Dysfunction in the vmPFC impacts its association with the amygdala, resulting in potentiated response to emotional cues (116). Non-treatment-seeking alcoholics have aberrant anterior insular cortex connectivity, a region associated with assignment of emotional states to interoceptive bodily stimuli (117). There is evidence of increased cortico-limbic connectivity in cocaine-dependent subjects, associated with emotion-based impulsivity (118). A model-free, resting-state study of alcohol-dependent subjects found increased within-network connectivity in salience, orbitofrontal, and default mode networks and increased between-network connectivity associated with higher self-reported ratings of negative urgency (119). These findings indicate interconnected, functionally coupled sets of brain regions associated with emotional activation and responding, which must be better understood to further development of more targeted treatment strategies. There are numerous avenues for further exploration using connectivity tools, which would immeasurably enhance insight into underlying mechanisms of behavior. Unfortunately, neuroimaging techniques are inherently difficult to conduct in animal models and may be confounded by agents used to anesthetize subjects, limiting investigations at the preclinical level.

### Human Neurotransmitter Systems

Function in many of the above brain regions is largely mediated by dopamine (DA) and serotonin (5HT) transmitter systems. These systems interact to influence emotion-based decision-making. Researchers have reported that low levels of 5HT are associated with greater incidences of rash or maladaptive behavior involving risk and increases in negative affect (120–125). Conversely, subjects with lower mono-amino oxidase, responsible for the breakdown of 5HT, display higher levels of aggression and negative urgency (126), which suggests that it may be the dysregulation of serotonin that influences emotional lability rather than simply a depletion. Interestingly, high 5HT levels in the PFC are correlated with socially adept behavior in monkeys (127), while low levels of 5HT in the anterior cingulate cortex are correlated with inhibited emotional regulation (124). It is important to note that these associations are receptor-dependent. The 5HT system is composed of several types of receptors and transporters, which may infer opposite effects upon activation. For instance, a reduction in 5HT available at 5HT_2c_ and 5HT_1a_ receptor sites increases likelihood of impulsive, rash behavior, while low 5HT at 5HT_2a_ receptor sites reduces risky behavior (128, 129). This dichotomy in receptor effects is important when determining possible pharmacological treatments. Although 5HT_2c_ and 5HT_1a_ receptors are more common, any prospective treatment targeting this system would be better served to attempt pharmacological effects specified for these receptors.

Alternatively, greater levels of DA activity are associated with a higher tendency to act (130), greater behavioral disinhibition (131), and greater reward-seeking and risk-taking behaviors (132, 133). DA is highly influential in the OFC–amygdala circuit (considered to be the “reward” circuit), particularly at D2 and D4 receptor sites, and it is theorized that DA effects on rash action may stem from D2 activation decreasing the value of delayed rewards (134). A recent study evaluating DA availability using positron emission tomography (PET) found that increased levels of DA in the putamen were associated with greater levels of impulsivity on the delay discounting task (135). The DA system in the OFC–amygdala circuit is modulated by serotonergic input through both direct and indirect mechanisms (136, 137). 5HT systems that subsume information processing influence DA pathways that underlie approach behavior (120, 138); therefore, decreased 5HT levels would result in diminished suppression of rash, ill-advised behavior.

### Human Genetics

Overall, it is well-established that behavior consistent with negative urgency is associated with depleted 5HT levels in the PFC and overactive DA in the OFC–amygdala circuit. There are a number of genetic polymorphisms possibly contributing to the imbalances observed in populations endorsing elevated negative urgency. Certain alleles of the serotonin transporter gene (5HTTLPR) and DA receptor genes (DRD2, DRD3, and DRD4) have all been associated with fluctuations in 5HT and DA levels and, thus, frequency of emotion-based rash behaviors (139–144). Associations between DA and 5HT are also present in an animal model of negative urgency. Yates et al. found that increased DA transporter function in the nucleus accumbens and greater 5HT transporter function in the OFC are mediated by higher exhibited negative urgency in a reward omission task (57). This neurobiological similarity highlights the usefulness of animal models to further elucidate the correlation between negative urgency and risky behavior, such as alcohol consumption.

There is limited information regarding the association between these neurotransmitter systems and negative urgency in alcohol use disorder populations. There are numerous lines of evidence indicating that decreased levels of 5HT are strongly associated with increased alcohol consumption and risk for future alcohol problems (145–147). One recent study reported that negative urgency (as measured by UPPS-P) mediated the relationship between alcohol abuse and genetically driven decreases in 5HT availability (148). In this study, higher polygenic 5HT scores (indicating lower 5HT function) were positively correlated with higher self-reported negative urgency and greater levels of alcohol consumption. At this time, there are no preclinical investigations evaluating the relationship between these transmitter systems, alcohol overconsumption, and negative urgency, although one study did report an increase in negative affect during cocaine withdrawal in rats in the reward omission task (58).

In addition to the influence of the DA and 5HT systems, there is evidence that polymorphisms of the GABRA2 gene, which codes for the GABA_A_a2 receptor, are also associated with both alcoholism risk and negative urgency (149). Villafuerte (150) identified an association between impulsiveness and alcoholism with genetic variants of the GABRA2 gene in a family strongly endorsing alcoholism (150). This same study reported that this association was mediated by activation during reward or loss. Further research uncovered that impulsiveness, particularly negative urgency, mediates the association between the GABRA2 gene and alcoholism (151). In addition, lower levels of gamma-Aminobutyric acid (GABA) in the dlPFC are correlated with greater reports of negative urgency (152). This region is implicated in the effortful control of behavior and is heavily activated during behavioral inhibition tasks in subjects high in negative urgency. Lack of GABA in this brain region may inhibit function, decreasing the ability of the dlPFC to efficiently regulate behavior.

### Implications for Treatment

Taken together, these findings provide excellent opportunities for translation to animal models, with the ultimate goal of improving clinical treatment. Translation of negative urgency to preclinical models would improve treatment in three main ways.

First, it allows for more precise identification of the neural correlates of negative urgency in human neuroimaging studies. Examining these regions translationally allows for more precision in the identified regions, circuits, and neurotransmitter systems. For example, identified neural substrates mediating negative urgency may be manipulated in animal models, and support of those hypotheses would provide data suggesting potential mechanisms through which those substrates may be manipulated to aid in treatment. Thus, this would help determine if these brain correlates are simply associated with negative urgency or are a causal mechanism in these maladaptive behaviors. This could lead to novel neurological and physiological targets.

Relatedly, animal models provide a unique opportunity to further elucidate neural underpinnings of behavior through manipulation of genetic predispositions. There are several lines of mice and rats selectively bred to prefer alcohol, including alcohol-preferring rats, high alcohol-drinking rats, and high alcohol-preferring mice (153, 154). Behavioral models can be used to compare responding to negative urgency in these subjects to responding observed in low alcohol-preferring subjects. These methods have demonstrated the ability to identify behavioral characteristics inherited alongside the predilection to prefer and consume alcohol, including impulsive-like responding in a delay discounting task (81, 155). Furthermore, they increase understanding of contributions of specific brain regions, networks, and neurotransmitter systems on alcohol consumption and associated risky behavior (156–160). Manipulation of brain region function in selectively bred animals through lesion studies, or neurotransmitter systems through pharmacological agents, grants researchers the ability to assess how each component affects behavior and determine what modifications may be employed to alter that behavior. Importantly, upon development of an animal model of negative urgency, researchers can better understand the relationship of negative affect and alcohol consumption. Although research implies that trait negative urgency contributes to the progression of alcohol use disorder, is it possible that prolonged heavy alcohol use increases the influence of negative affect? As noted above, research in humans suggests that the immediate relief from negative emotions provided by alcohol consumption increases the likelihood of repeated pairings. Animals that are selectively bred to prefer alcohol grant researchers the ability to evaluate this premise and manipulate factors, such as neurotransmitter systems, which facilitate this association. The use of selectively bred animals is recommended when investigating the interaction of two traits (alcohol preference and negative urgency).

Second, it enables the testing of novel compounds and their ability to reduce negative urgency-like behaviors, which, if successful, could then be applied and tested in clinical models. This treatment may be pharmacological, as in a drug purported to reduce anxiety (targeting the affective aspect), or behavioral, such as training the subject to reduce excessive lever pressing in times of stress (targeting the rash behavior aspect). Examining these treatments in animals first allows for testing of initial feasibility, safety, and effectiveness prior to implementing such interventions in humans. These treatments may produce objective, quantifiable outcomes that can then be administered in a clinical setting. Given the demonstrated influence of negative urgency on increased alcohol consumption in alcohol use disorder populations and the increased elucidation of neural mechanisms underlying this association, developing novel therapeutic targets through animal models should constitute the next step toward more efficacious treatment options. Upon design of a translational, externally valid animal model, there are numerous possible targets of investigation. In particular, administration of agents designed to boost 5HT availability, specifically in the PFC, could generate a cascading effect downstream in the DA system of the OFC–amygdala circuit. Alternatively, increasing levels of GABA in the dlPFC may heighten the ability of this region to function efficiently, alleviating the heavy cognitive load required by subjects with high negative urgency to inhibit behavior.

Translation to animal models in this way has provided greater understanding of neural correlates of behavior in several domains. For instance, the symptoms of bipolar disorder are often alleviated through lithium administration, which also serves to decrease incidents of suicidal behavior (161–163). Valproate, an alternate method for bipolar disorder treatment, is also efficacious in relieving symptoms, but has no effect on suicidal behavior (164). A study evaluating the effects of these drugs on impulsivity using the delay discounting task revealed that lithium was more effective at reducing impulsive behavior in that paradigm, which may underlie the decrease in suicide attempts in that population (165). Models of Parkinson’s and Alzheimer’s disease have provided valuable information on the neural deterioration or malfunctioning that accompany the symptoms of those disorders (166–170). Animal models of depression have helped identify brain network and neurotransmitter systems associated with negative affect (171–174). One of the most useful tools of the animal model is to administer pharmacological therapies to attempt to identify what neurobiological mechanisms underlie symptoms and behavior.

Third, it allows for the manipulation of negative urgency in human studies and how changing the immediate expression of the trait can be clinically applied. Such studies would help test the viability of potential interventions in changing negative urgency in the human laboratory. It would also allow the use of a behavioral task of negative urgency as an objective marker of change for clinical trials of negative-urgency-based interventions, avoiding limitations related to self-report, as described above. Therefore, it is clear that the use of objective translational paradigms of negative urgency would be conducive to advancing research concerning the treatment of addictive disorders, as well as other comorbid disorders related to negative urgency.

## Conclusions, Limitations, and Suggestions for Future Work

We propose two important gaps in research on negative urgency that should be filled as next steps in the long-term goal of intervening on negative urgency. First, existing translational approaches for negative urgency only assess negative affect or impulsive behavior, but not both. We suggest that only by combining these two aspects of negative urgency into one model can we increase the validity of the model in both animal and human subjects. There is still some work to do to figure out the most valid way to create such a translational model. In devising a translatable method to accurately evaluate negative urgency, and more importantly to develop a model that is sensitive enough to detect changes in behavior, investigators should seek to preserve as many aspects of validity as possible (**Table 1**). Of course, other potential methods could be developed, but should meet minimal validity criteria. Importantly, many of the methods of inducing negative emotions in humans have limited translatability, hindering the design of a truly translational task. The reward omission task may be a prime place to start to induce negative emotion across human and animal models. However, other approaches to induce negative affect translationally may need to be species specific. For example, food restriction might be effective and useful in animal models, but these methods have feasibility and ethical restraints in human work. In an animal model, acute restraint stress, food deprivation, and foot shock are demonstrably effective at inciting negative affect; however, these methods may lead to increased corticotrophin levels or persistent depressive-like symptoms, so exposure should be minimal. Any combination of these methods should be thoroughly vetted and rigorously tested to establish both construct and predictive validity. In contrast, several of the impulsivity methods are highly translatable (**Table 1**) and serve as good starting points in designing a new model. Of these impulsivity tasks, the delay discounting task would be the most effective for use of cognitive impulsivity inquiries, while the Go/No-Go task would be most informative for measures of motor impulsivity.

One limitation of investigations of negative urgency is the difficulty in parsing specific emotional reactions for evaluation. There is inherently a great deal of overlap in experience of emotion; anger, fear, and sadness often co-occur and the neural underpinnings of singular emotions are highly interconnected. An excellent review from Price (175) on the neurocircuitry of mood disorders describes this phenomenon very succinctly, identifying several structures, including the primary structures of the limbic system and hippocampal regions, which contribute to various emotions and how those structures interconnect. Although the ability to efficiently unravel such closely related emotions (sadness, stress, etc). would be ideal, it has not yet been successfully accomplished and negative urgency has been shown and theorized to relate to multiple different negative emotions, including sadness, stress, and anger. Unfortunately, the inability to untangle specific emotions may hinder the ability of animal research to completely model the human experience.

Another important caveat of developing a translational model of negative urgency is the distinction between state and trait behavior, which show little overlap (46). This might limit the feasibility of developing a translational model. Current self-report evaluations of negative urgency in humans are effective at assessing trait levels of negative urgency; a similar trait-like construct may be modeled in animals through selective breeding. For example, the high alcohol-preferring mouse lines have demonstrated higher impulsive behavior in the delay discounting task (76), making them an ideal model for investigations of trait impulsivity. State behavior is successfully measured in both human and animal models, allowing for the potential of strong concordance between these groups. Although state and trait approaches do not overlap strongly, researchers propose that increased overlap would likely occur through increased specificity in the measures (46). For example, performing a behavioral task under a negative emotional state would theoretically lead to larger correlations between the state measure and the trait of negative urgency.

Second, research has yet to examine identified human neural and genetic correlates of negative affect in animal models. There are numerous avenues for exploration that are well supported in the field and should be manipulated in animal models to test their potential treatment value. Abundant research implicates dysfunction of the serotonin and dopamine systems in regulation of the OFC–amygdala circuit may contribute to behavioral disinhibition during negative emotional states. There is also evidence of the contribution of GABA unavailability in the PFC, which may account for the excessive activation during response inhibition in subjects high in negative urgency. Identifying agents that efficiently reduce impulsive behavior during negative urgency in an animal model provides a basis for potential applications in a clinical setting. Future work should aim to further elucidate the influence of the OFC–amygdala circuit (emotional processing) and regions of the prefrontal cortex (behavioral control) in negative urgency. Novel pharmacological treatments can be discovered through manipulation strategies only available in animal models, which can then be applied in a clinical setting. It should be noted that although several lines of research have identified rodent brain regions as homologous to the human prefrontal cortex regions, there is no definitive nomenclature that accurately confirms these regions (169), which may limit translatability of neural data across preclinical and clinical models, although this type of work has been successful in related disorders (for review, see Ref. 93).

In conclusion, we suggest that future studies should seek to devise and test a valid translational model of negative urgency that is easily administered in both animals and human subjects. We hope that our review not only answers some questions about how to do this, but also creates new questions that can improve and advance this work more in the future. The long-term goal of such work will be to bring together basic research on negative urgency and clinical practice of addiction medicine, for more effective prevention, treatment, and rehabilitation of addictive disorders. We suggest that development of this model will allow for determination of prime neurological and physiological treatment targets, the testing of treatment effectiveness in the preclinical and the clinical laboratory, and, ultimately, improvement in negative-urgency-related treatment response and effectiveness. Given that negative urgency is a transdiagnostic risk factor that impedes treatment success, the impact of this work could be large in reducing client suffering and societal costs.

## Author Contributions

All authors contributed to the overall direction and goals of this review. The original concept was generated and broadened by MC, who was also responsible for edits and comments. EA wrote the introduction to urgency, outlined research in clinical subjects using the UPPS self-report measure, and contributed to editing and comments. MH wrote the bulk of the paper, including sections on animal models and possible neural correlates for investigation and contributed to overall edits and comments.

## Funding

Indiana University Grand Challenge Project; Responding to the Addiction Crisis (no grant number) R01AA027236 P60 AA007611 to Melissa Cyders.

## Conflict of Interest Statement

The authors declare that the research was conducted in the absence of any commercial or financial relationships that could be construed as a potential conflict of interest.
